# Efficacy of a Novel *Lactiplantibacillus plantarum* Strain (LP815^TM^) in Reducing Canine Aggression and Anxiety: A Randomized Placebo-Controlled Trial with Qualitative and Quantitative Assessment

**DOI:** 10.3390/ani15152280

**Published:** 2025-08-04

**Authors:** Emmanuel M. M. Bijaoui, Noah P. Zimmerman

**Affiliations:** 1 Argos Bio LLC, 169 Madison Ave., New York, NY 10016, USA; 2 Verb Biotics LLC, 27 Drydock Ave., 8th Floor, Boston, MA 02210, USA; noahz@verbbiotics.com

**Keywords:** canine behavior, dog anxiety, aggression in dogs, GABA-producing probiotics, *Lactiplantibacillus plantarum*, gut–brain axis, microbiome modulation, companion animals, activity monitoring

## Abstract

Behavioral issues in domestic dogs represent a significant welfare concern affecting both canines and their caregivers, with prevalence rates reported to range from 34 to 86% across the population. Current treatment options, including selective serotonin reuptake inhibitors (SSRIs) like fluoxetine, often present limitations including adverse effects and delayed efficacy. This randomized, placebo-controlled (maltodextrin) study investigated the effects of a novel *Lactiplantibacillus plantarum* strain (LP815^TM^) on canine behavioral concerns through gut–brain axis modulation. Home-based dogs (n = 40) received either LP815^TM^ (n = 28) or placebo (n = 12) daily for 4 weeks, with behavioral changes assessed using the comprehensive Canine Behavioral Assessment & Research Questionnaire (C-BARQ) and continuous activity monitoring. After the intervention period, dogs receiving LP815^TM^ showed significant improvements in aggression (*p* = 0.0047) and anxiety (*p* = 0.0005) compared to placebo controls. These findings were corroborated by objective activity data, which demonstrated faster post-departure settling, reduced daytime sleep, and improved sleep consistency in the treatment group. Throughout >1120 administered doses, no significant adverse events were reported, contrasting favorably with pharmaceutical alternatives. The concordance between our findings and previous research using different *L. plantarum* strains suggests a consistent biological mechanism, potentially involving GABA production and vagal nerve stimulation. These results indicate that LP815^TM^ represents a promising, safe alternative for addressing common canine behavioral concerns with potential implications for improving both canine welfare and the human–animal bond.

## Simple Summary

Dogs struggle with anxiety and aggression, but treatment options can be limited or come with undesirable side effects. This study tested a new probiotic strain, *Lactiplantibacillus plantarum* LP815^TM^, to see if it could naturally support better behavior in dogs. Over 5 weeks, dogs who received the probiotic showed improvements in anxiety and aggression, slept more regularly, and adjusted more easily when their owners left home. No side effects were reported. These findings suggest that this probiotic may help dogs feel calmer and behave more consistently by supporting gut health.

## 1. Introduction

Behavioral concerns in domestic dogs represent a significant welfare issue impacting both canines and their caregivers. Epidemiological studies report prevalence rates from 34 to 86% in companion dogs, with fear and anxiety consistently among the most frequent concerns [1,2,3]. A large Finnish study (n = 13,715) found that 32% of dogs exhibited noise sensitivity and 29% displayed general fearfulness [4]. These behavioral issues—primarily fear, anxiety, aggression, and noise sensitivity—compromise canine welfare and create emotional, social, and financial burdens for caregivers [5]. Significant comorbidity exists between different behavioral concerns, with Dinwoodie et al. (2019) finding that over half of dogs displaying aggression also exhibited fear or anxiety [3]. Unlike human psychiatric conditions with standardized diagnostic criteria, canine behavioral issues are typically classified through broad categories based on observed responses rather than biological markers [6,7,8,9,10,11,12,13,14].

The rise in canine aggression poses risks to caregivers and public safety, with the UK recording a 51% increase in dog attacks between 2018 and 2023 [15]. Contributing factors include urbanization and pandemic-related changes in human–dog interactions. Recent research has identified associations between disease status and fear/aggression behaviors, suggesting possible immune or microbiome-mediated pathways [1]. The high prevalence and impact of these issues underscore the need for effective, evidence-based interventions.

For severe behavioral concerns, veterinarians often prescribe medications such as selective serotonin reuptake inhibitors (SSRIs) [16]. While the commonly used SSRI fluoxetine (Prozac^®^ Eli Lilly and Company, Indianapolis, IN, USA. Reconcile^®^ Pegasus Laboratories, Inc., Pensacola, FL, USA) can be effective for managing canine anxiety, aggression, and compulsive behaviors, it may present side effects including reduced appetite and lethargy in some dogs [17,18,19]. Additionally, pharmacological interventions may require ongoing veterinary administration, including added cost and issues with accessibility, to maintain effectiveness, highlighting the need for complementary approaches [20,21,22]. Commercial natural supplements (chamomile, *L-Theanine*, *L-tryptophan*) lack rigorous testing for long-term efficacy and side effects [23,24,25,26]. Emerging research has focused on the role of the gut microbiome—the population of microorganisms in the gastrointestinal tract that contribute to host health through bioactive metabolites—in modulating behavior via the gut–brain axis [26,27,28].

Gamma-aminobutyric acid (GABA), a metabolite produced by gut bacteria, has been shown to modulate anxiety, epilepsy, and depression in humans and canines [29]. Although GABA itself crosses the blood–brain barrier (BBB) inefficiently, it can increase brain-produced GABA through two main mechanisms: stimulation of the vagus nerve, prompting further GABA production in the brain; and production of metabolites that cross the BBB and act as precursors to GABA [30]. Additionally, GABA reduces gastrointestinal inflammation and gut permeability, preventing inflammatory agents from triggering cascading events.

*Lactiplantibacillus plantarum* (LP815^TM^), a novel probiotic strain recently shown to increase GABA levels in the body [31], offers potential for sustained native GABA production through gut microbiome modulation. Human trials demonstrate significant positive impacts on behavioral conditions and sleep quality. Given the overlap in symptoms and biological pathways between humans and canines, and the lack of suitable alternatives for canine behavioral issues, exploring LP815^TM^’s potential in dogs is of significant importance. This study was designed to reflect real-world conditions, recruiting home-based dogs of various breeds, ages, and locations to assess the probiotic’s effectiveness in their natural environment.

## 2. Materials and Methods

This interventional study evaluated the effects of a daily lactic acid probiotic supplement, *Lactiplantibacillus plantarum* 815 (LP815^TM^), on canine behavior and general health parameters over a 4-week (28-day) daily feeding regimen. Home-based subjects (n = 40) completed standardized assessments on days 0 and 35, prior to any administration of the supplement as well as upon completion of the 4-week feeding regimen. The study spanned 5 weeks, with the initial 7 days acting as a baseline period with no intervention to properly calibrate the wearable equipment as well as assess behavior monitored by the questionnaire. A written study protocol was finalized before enrollment; the document was kept on file but was not preregistered in a public repository.

To successfully assess the impact of supplementation, 30% of the cohort (n = 12) was provided a placebo supplement in order to adequately contrast the impact of the treatment. This exploratory study was pragmatically sized at 40 dogs and powered only informally; the primary outcome pre-specified for hypothesis testing was the C-BARQ aggression composite score. A multimodal assessment strategy of behavior analysis was employed, using both qualitative and quantitative methods as detailed below.

The sample size for this trial was determined using an a priori power analysis for a two-tailed independent *t*-test (α = 0.05). As this is the first trial to explore the use of this strain to modulate canine behaviors, we assumed a conservative large effect size (Cohen’s d = 0.7), based on prior studies using C-BARQ changes as an endpoint in dogs with behavioral concerns. Using this, a total of 34 dogs (24 treatment, 10 placebo) was estimated to achieve 70% power. To enhance statistical power, we randomized 40 dogs (28 treatment, 12 placebo), yielding 80% power to detect an effect of d = 0.7 and 47% power for a medium effect (d = 0.5), with 70% power achieved at d ≈ 0.64. Given the biological plausibility of moderate-to-large effects in this population as well as statistically significant outcomes in the human parallel of this study, the sample size of 40 dogs was deemed sufficient a priori to detect clinically meaningful differences in C-BARQ scores.

### 2.1. Recruitment

Participants were recruited from Treat Therapeutics’ clinical research database to represent the target consumer demographic. Inclusion criteria specified companion dogs aged 1–12 years residing in the United States, with no restrictions on baseline gastrointestinal status, breed, size, or diet type to ensure representative sampling of the target population. While many commercial diets presently claim to contain pre- and probiotics, we did not exclude participants that were fed these diets for several reasons. Firstly, due to the wide range of regulations and quality control around these diets, it is hard to assess which of the claimed ingredients are present and active at the claimed inclusion levels. Second, participants were enrolled precisely because existing interventions or regular choices had no impact on the behavioral conditions of their dogs.

Dogs were recruited based on owner-reported behavioral concerns, with intentionally broad inclusion criteria regarding the type of concern (e.g., aggression, anxiety) due to the high comorbidity among canine behavioral conditions and uncertainty about which specific behaviors might be most responsive to supplementation. Due to the decentralized nature of the study, participants were enrolled on a rolling basis in several distinct waves. Each participant received a canine-specific activity monitor (FitBark 2, Dog Activity Monitor, FitBark Inc., Kansas City, MO, USA.), a 4-week supply of either the LP815^TM^ or a maltodextrin placebo according to their cohort assignment, and printed guidance materials detailing study procedures. All 40 participants met predetermined screening criteria and provided informed consent for study participation and data utilization. These inclusion/exclusion criteria were defined a priori; no additional animals or data points were removed post hoc.

### 2.2. Study Conduct and Management

This home-based investigation employed standardized delivery of study materials to participants’ verified locations. Dosage followed manufacturer guidelines based on subject weight, with each capsule (containing 1 billion CFU of LP815^TM^) administered per 25 lbs of canine weight. Dogs below 25 lbs were provided with the minimum dose of 1 capsule. In vitro experimentation of LP815 has shown that 1 billion CFU produces 100 mg of GABA within a 24 h period in gut-like conditions. This is well below the safety characterization limits in dogs, with chronic administration of up to 1 g/kg/day showing no toxicity or adverse effects [32]. Gabapentin, a structural analog of GABA, is routinely administered to dogs for anxiety concerns, with dosage levels of 20–25 mg/kg up to 2–3 times daily, once again significantly lower than the expected production capacity of LP815 [33,34,35]. In a parallel human study, LP815 was demonstrated to reduce mild-to-moderate anxiety in generally healthy patients [36]. Whole-genome sequencing confirms that LP815 belongs to *Lactiplantibacillus plantarum*, and the strain has been deposited in the ARS-NRRL culture collection. Comprehensive genomic and phenotypic safety profiling showed no transferable antimicrobial-resistance or toxin genes. An independent expert panel has accordingly granted LP815 self-affirmed GRAS status [37].

The maltodextrin placebo was identically prepared and dosed, maintaining participant blinding to cohort assignment. Food allergy declarations were collected from pet caregivers prior to study initiation as a safety precaution, although the test product contained only the probiotic and bulking agents.

Behavioral changes were assessed through both quantitative and qualitative methods. All participants completed the standardized Canine Behavioral Assessment & Research Questionnaire (C-BARQ), developed by the University of Pennsylvania [38]. This validated instrument was selected for its comprehensive evaluation of canine behavior, having already been used to assess over 50,000 dogs across 300 breeds and cross-breeds. The C-BARQ provides distinct categorization of various behavioral domains, including training and obedience, aggression, fear and anxiety, separation anxiety, excitability, attention-seeking, and miscellaneous negative behaviors. The 100+ question assessment was administered at baseline (day 0) and study conclusion (day 35). Additional data collected included biometric information (breed, body weight, height), diet, concurrent supplement use, fecal characteristics (color and consistency), pre-existing health conditions, and activity levels. Weekly follow-up surveys monitored adverse events and environmental changes that might confound study outcomes.

Participants were randomized to either the active treatment (n = 28) or placebo (n = 12) groups. A simple 70:30 allocation list was created with the Excel Rand() function (seeded at study start); dogs were assigned in enrollment order. While pet caregivers remained blinded to assignment, complete investigator blinding was not possible due to adverse event monitoring requirements. Importantly, participants were not pre-selected based on any criteria beyond the established inclusion parameters.

Upon receipt of study materials, participants completed the baseline C-BARQ assessment and attached the provided activity tracker (FitBark 2, Dog Activity Monitor) to their dog’s collar. The activity monitor was linked to the corresponding FitBark mobile application, with researcher access enabled for real-time data collection. A 7-day baseline monitoring period preceded supplementation to establish activity and sleep patterns and monitor for any unexpected events. Subsequently, pet caregivers administered the daily supplement either by opening the capsule and mixing the powder with food or by direct pill administration when feasible. Participants were instructed to refrigerate the supplement to maintain probiotic viability. No financial incentives were provided beyond the study materials; participation was motivated by caregivers’ interest in addressing their pets’ pre-existing behavioral conditions. Protocol adherence was monitored through direct participant engagement and continuous FitBark dashboard observation [39].

All dogs remained in their own homes, eliminating cage-location effects. Each dog completed a 7-day baseline run-in before supplementation, serving as its own control. Allocation was fixed (no cross-over), preventing order effects. C-BARQ was completed exclusively on day 0 and day 35; activity data were collected continuously. Longitudinal activity analyses used a mixed-effects model in the statsmodels python library (V0.14.4) [40] with dog ID as a random effect to account for residual inter-dog variation.

The experimental time period and design of the study were based on existing norms and research specific to the canine microbiome. Notably, several studies have shown that the canine microbiome rapidly shifts within a matter of days [41,42]. In supplement-specific studies, there exists evidence that a 4-week interventional period is sufficient to observe statistically significant responses in both microbiome profiles and behavioral indicators [41,42,43,44,45].

### 2.3. Study Cohort

Following established recruitment practices, we enrolled 50 dogs initially to account for an anticipated 20% attrition rate. Despite multiple pre-study contact points designed to establish participant rapport, 10 subjects became unresponsive prior to study commencement and were excluded from the protocol. The remaining 40 subjects completed the entire 35-day protocol, yielding an 80% completion rate. As none of the non-responsive participants completed the initial survey, adverse events are unlikely to have contributed to their withdrawal from the study. Given that different behavioral conditions may overlap, participants were able to select multiple behavior conditions upon onboarding the final cohort, as well as a free-text section allowing pet caregivers to highlight specific conditions not addressed by the categories provided. Aggression (26%), social anxiety (23%), and separation anxiety (20%) were the conditions most frequently tagged by the participants, with others (vocalization issues, reactivity, destructive behavior, general anxiety, panic attacks, fearfulness, specific phobias, hyperactivity, and dominance behaviors) all representing less than 10% of frequency each, and likely overlapping in definition with the other categories provided.

### 2.4. Activity Monitor Data

Activity data were collected continuously via the FitBark 2 Dog Activity monitor. The device provided minute-level resolution activity data, categorizing canine movement patterns into “rest”, “active”, and “play” states with increasing activity level thresholds. Categorization thresholds were established by FitBark using proprietary algorithms based on thousands of hours of canine activity data. All data were automatically transmitted to a centralized web dashboard, allowing the research team to monitor and extract participant-level activity information in CSV format. Data files were generated for each participant at multiple temporal resolutions (minute, hourly, and daily aggregations). The activity monitoring devices were purchased directly from FitBark, and no commercial relationship or financial incentives existed between the research team and FitBark in relation to this study.

### 2.5. C-BARQ Assessment

As described above, the 100-question C-BARQ assessment was used to quantify behavioral changes observed post-supplementation (comparing day 0 to day 35 results). C-BARQ scores are segmented into distinct behavioral categories, and analysis was conducted according to these established domains (training and obedience, aggression, fear and anxiety, separation anxiety, excitability, attention-seeking, and miscellaneous negative behaviors).

Each question aims to identify a specific trait associated with negative behavioral effects (e.g., “Your dog is approached directly by a household member while s/he is eating—how do they respond?”) and includes 5 response options assessing frequency and intensity of the observed trait (e.g., Serious aggression, Aggression, Moderate aggression, Mild aggression, No aggression, NA/Never Observed). For analysis purposes, positive traits were assigned higher numerical values (i.e., No aggression = 5), whereas more negative traits were assigned lower values (i.e., Serious aggression = 1). Responses marked as “NA/Never Observed” were assigned a value of ‘0’ and excluded from statistical analysis using standard protocols for missing data handling within the NumPy Python library (1.26.3).

This validated assessment tool was selected for its methodological robustness and established reliability in canine behavioral research [37]. The research team accessed the C-BARQ assessment independently, and no institutional or financial relationship existed between this study and the University of Pennsylvania School of Veterinary Medicine where the assessment was developed.

### 2.6. Post-Analysis

All data analysis was performed in a Python-based Jupyter notebook within the Google Colab environment (Python 3.11.11 with Jupyter Notebook 6.5.5, google-colab v1.0.0). Standard statistical and data processing libraries were employed, including Pandas (2.2.3), NumPy (1.26.3), Seaborn (0.13.2), and SciPy (1.11.4) for data processing and statistical testing.

Normality of each continuous outcome was assessed with the Shapiro–Wilk test and Q–Q plots; homogeneity of variances between groups was checked with Levene’s test. When assumptions were met, two-sample *t*-tests were applied; otherwise, the Wilcoxon rank-sum test was used. Mixed-effects-model residuals were visually inspected for normality and homoscedasticity. All tests were two-sided, with *p* < 0.05 deemed statistically significant.

No specialized bioinformatic workflows were required for this study.

## 3. Results

This study represents the first comprehensive evaluation of this supplement in a large, diverse canine cohort, designed to provide critical insights for potential mass-market application. While preliminary safety evaluations had been conducted in small-scale human and canine trials, this investigation sought to assess potential adverse effects in a larger, more diverse canine population. Concomitantly, the study analyzed both standardized behavioral assessment data (C-BARQ) and objective activity metrics to determine whether sustained supplementation significantly impacted canine behavioral parameters.

The final cohort (n = 40) comprised 23 distinct breeds, with mixed-breed dogs representing the largest proportion across both study groups. Age distribution reflected expected population demographics, with a median age of 4.6 years. Gender distribution was 67.4% male (n = 27) and 32.5% female (n = 13), with 87.5% (n = 35) of all dogs being neutered. This high neutering rate suggests that the behavioral issues observed were persistent despite this common intervention for temperament regulation. Dietary regimens varied among participants, including raw, standard dry food, air-dried, fresh, and mixed diets, with the majority (55%, n = 22) consuming standard dry diets.

### 3.1. Adverse Event Monitoring

Adverse events in canine studies typically center on gastrointestinal disturbances, particularly emesis (vomiting) and diarrhea, which can result from probiotic dosage intolerance or general product sensitivity. Our study protocol included weekly adverse event monitoring, with collection of up to 200 total data points (5 timepoints × 40 participants). We observed moderate response compliance with interim surveys (Days 7, 14, 21), collecting 146 data points (73% completion rate).

Emesis events were reported only 13 times throughout the study period, representing 8.9% of all collected survey responses. Seven of these events occurred in the placebo group, and no statistically significant association was observed between supplement administration and vomiting. Temporal analysis revealed a slight increase in emesis events in the active treatment group on day 35 (n = 3), but this isolated finding without corresponding earlier reports likely represents sporadic occurrences rather than treatment-related adverse events.

**Figure 1:**
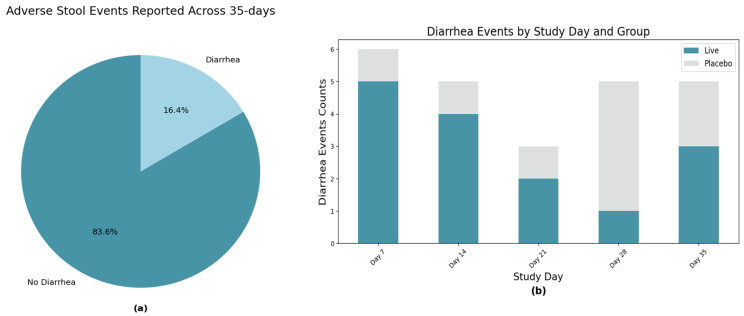
(**a**) Diarrhea events (16.4%) were more prevalent across the study than emesis events (8.9%) but still do not represent a significant adverse effect by the LP815 supplement. (**b**) When plotting for day-specific occurrences, diarrhea events on day 35 of the study were lower than on day 7 (post-baseline) for the live cohort, suggesting minimal negative impact on GI health.

In the study, diarrhea events were similarly infrequent, with only 24 reports (16.4% of collected data points) indicating substantial deterioration in stool quality. No significant differences in diarrhea incidence were observed between treatment and placebo groups across the 35 days (*p* = 0.81). Interestingly, we noted a general decrease in diarrhea events over the 35-day study period in the Live group, potentially suggesting a stabilizing effect of the probiotic on gastrointestinal function. However, as improved stool quality was not a primary endpoint, further targeted research would be necessary to substantiate any beneficial effects on existing gastrointestinal disturbances (Figure 1).

### 3.2. C-BARQ

As detailed in the methods section, the C-BARQ served as the primary instrument for assessing behavioral trait changes throughout the study, with higher scores indicating improvement in specific behavioral categories. To evaluate domain-specific changes, responses within each behavioral category were averaged per participant and then compared between cohorts, analyzing outcomes for Training & Obedience, Aggression, Fear & Anxiety, Separation Anxiety, Excitability, Attention Seeking, and miscellaneous negative behavior traits.

**Figure 2:**
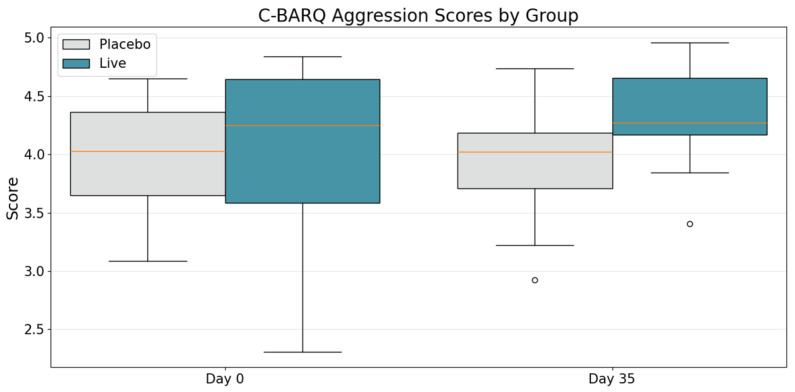
C-BARQ Aggression scores at baseline (day 0) and after 4 weeks of supplementation (day 35) in placebo-treated dogs (gray) and LP815™-treated dogs (green). At baseline, the groups were comparable (Welch *t* = 0.22, *p* = 0.83). By day 35, LP815™ dogs had higher (i.e., less aggressive) scores (median 4.28, SD 0.36) than placebo dogs (median 4.04, SD 0.56); the mean difference was +0.48 points on the 1-to-5 scale (95% CI 0.11–0.86, t_38_ = 2.76, *p* = 0.015). The within-group rise from baseline in LP815™ dogs trended toward significance (*t* = −1.60, *p* = 0.12). Box-and-whisker plots depict medians, inter-quartile ranges, and full data ranges for each time-point and treatment.

We observed significant improvement in aggression-related traits between the placebo and treatment cohorts at day 35, with median scores of 4.04 and 4.28, respectively (Figure 2, *t* = 2.760, *p* = 0.015). Within the treatment group, the difference between baseline and day 35 trended toward statistical significance (*t* = −1.60, *p* = 0.12), suggesting a positive but not significant effect of supplementation on owner-reported canine aggression. Similarly, the “Fear & Anxiety” category demonstrated statistical significance after 35 days of supplementation. Dogs in the treatment arm exhibited significantly higher scores (indicating lower fear and anxiety traits) at day 35 compared to placebo dogs, with median values of 4.26 and 3.19, respectively (*t* = 3.28, *p* = 0.0052). Longitudinal comparison within the treatment group revealed an increase in median score from 3.92 to 4.26, trending toward statistical significance (*t* = −1.81, *p* = 0.078).

**Figure 3:**
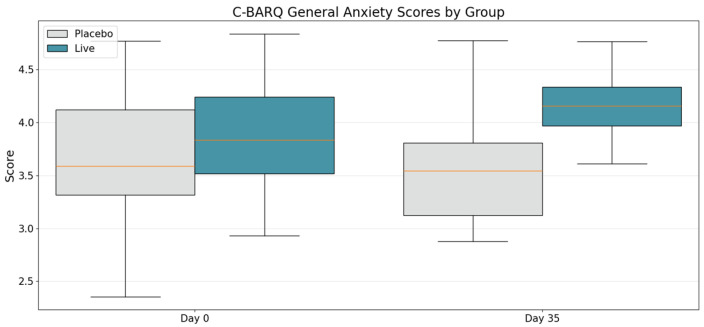
C-BARQ General-Anxiety scores at baseline (day 0) and after 4 weeks of supplementation (day 35) in placebo dogs (gray) and LP815™ dogs (green). The groups were comparable at baseline (Welch *t* = 0.99, *p* = 0.34). By day 35, LP815™ dogs recorded higher (i.e., less anxious) scores than placebo dogs; the mean difference was 0.46 points on the 1-to-5 scale (95% CI 0.08–0.83; Welch t_38_ = 2.59, *p* = 0.022). Within the LP815™ group, scores rose significantly from baseline (paired *t* = −2.66, *p* = 0.011).

Given the well-documented overlap between behavioral conditions described in the introduction, and the presence of both “Fear & Anxiety” and “Separation Anxiety” categories in the C-BARQ assessment, an exploratory analysis was conducted combining these domains into a “General Anxiety” measure. This approach, which also helped address potential data limitations from “N/A” responses due to question specificity, revealed stronger statistical significance. The combined analysis (Figure 3) demonstrated significant improvements in general anxiety between the placebo and treatment arms at day 35 (*t* = 2.59, *p* = 0.022), as well as a significant within-group improvement in the treatment arm from baseline to day 35 (*t* = −2.66, *p* = 0.011). These findings suggest that the supplement effectively modulated both general anxiety and aggression in dogs within the 4-week administration period.

While changes were observed in other assessed categories (Training & Obedience, Excitability), these did not reach statistical significance between treatment and placebo arms at day 35, suggesting that the supplement’s effects may be most pronounced for anxiety and aggression-related behavioral traits.

Moreover, a detailed item-level analysis of C-BARQ responses revealed that among the treatment cohort, the 10 questions showing the largest changes between baseline and day 35 were predominantly related to aggression (7 out of 10). In contrast, the placebo cohort demonstrated more heterogeneous changes, with questions related to “Negative behaviors” and “Attention seeking” categories showing the most notable shifts (3 out of 10 each) (Supplementary Figure S1). This pattern further substantiates the finding that the supplement had a targeted effect on specific behavioral domains, particularly aggression, while placebo responses were more randomly distributed across behavioral categories. These differential response patterns reinforce the validity of the study design, which included both treatment and placebo groups. They also strengthen the conclusion that the observed improvements reflect specific treatment effects rather than nonspecific changes or reporting bias.

### 3.3. Activity Tracker Data

Wearable activity monitors provided continuous, high-resolution data on canine behavioral patterns throughout the study period. The devices captured data at multiple temporal resolutions, from minute-level to daily summaries, enabling comprehensive analysis of behavioral shifts in both treatment and placebo cohorts. Given the continuous nature of these measurements, we implemented several data processing protocols to enhance signal detection. These included excluding weekend activity data to control for expected increases in activity associated with greater owner availability. While certain confounding variables (e.g., weather-related activity fluctuations) could not be fully accounted for due to dataset limitations, the longitudinal depth and temporal resolution of the activity data provided a robust framework for evaluating supplement efficacy in modulating behavioral trends.

**Figure 4:**
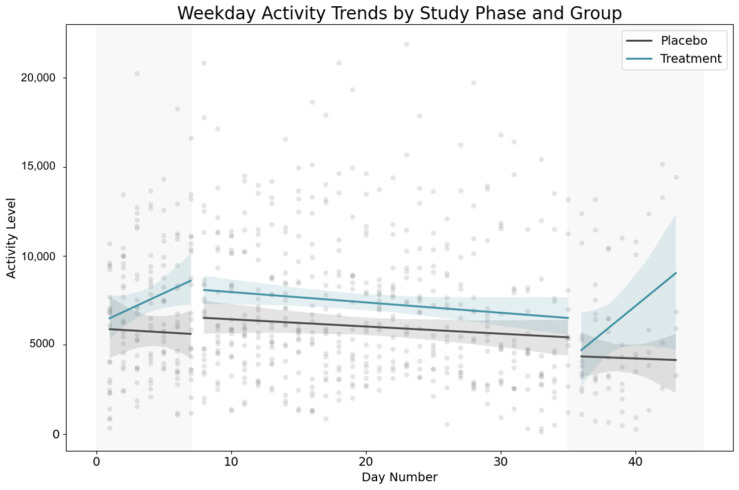
Weekday activity trends (FitBark units, u) for placebo (gray) and LP815™ (green) dogs, with mixed-effects regression lines and 95% confidence bands. At baseline, the groups were similar (mean ± SD: LP815™ 5923 ± 1232 units; placebo 5847 ± 1219 u). Over the full study, LP815™ dogs accumulated a −57.8 units per day change relative to placebo (95% CI −120.5 to +4.9 u; z = −1.80; *p* = 0.069). Phase-stratified models gave Day × Group interaction slopes (coef., 95% CI u day − 1): pre-intervention +394.9 (−1.5 to 791.4), during −16.5 (−86.5 to 53.5), post +205.1 (−530.5 to 940.7). Although the overall effect narrowly crossed zero, the downward point estimate supports a progressive reduction in activity—corroborating the behavioral calming seen on C-BARQ.

Longitudinal analysis of activity patterns revealed differential trends between treatment and control groups throughout the study period. Dogs receiving the active supplement demonstrated a progressive decline in overall activity, with a slope of −27.59 activity units per day, contrasting with the placebo group’s slight positive slope of +18.87 units per day (Figure 4). While these isolated trends did not reach statistical significance in simple regression models (*p* = 0.331 and *p* = 0.566, respectively), the hierarchical structure of our data necessitated more sophisticated analytical approaches. To account for repeated measurements from the same subjects and control for individual variability, we implemented a mixed-effects model with dog identity as a random effect. This analysis revealed that treatment dogs experienced a daily activity decrease of −57.78 units relative to placebo dogs, a difference approaching statistical significance (*p* = 0.069).

**Figure 5:**
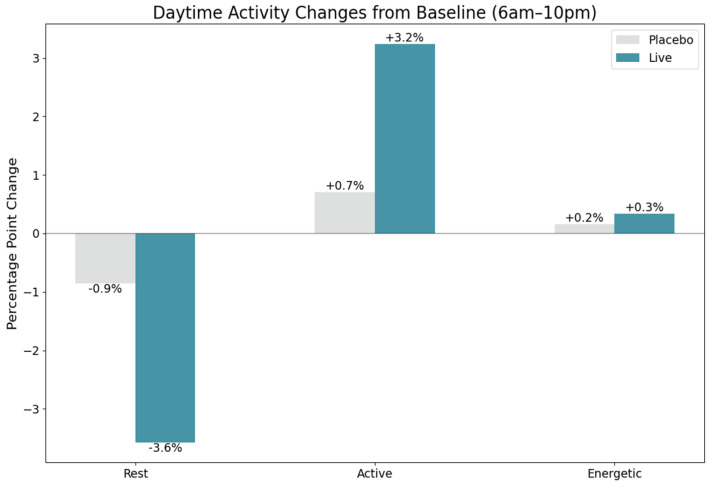
Daytime activity composition changes from baseline (6:00 AM–10:00 PM) in placebo (gray) and treatment (green) groups. Treatment dogs exhibited a greater reduction in daytime sleep (−3.6% vs. −0.9%) and a larger increase in moderate-intensity activity (+3.2% vs. +0.7%), with minimal differences in high-intensity activity. These patterns suggest improved wakefulness and balanced activity rather than sedation or lethargy.

Importantly, this reduction in overall activity did not compromise canine welfare or indicate sedation. Granular analysis of minute-level data during daytime hours (06:00–22:00) revealed changes in activity composition that further clarified the nature of the observed effects. Treatment group dogs exhibited a 3.6% decrease in daytime sleep, compared to a 0.9% decrease in the placebo group, suggesting improved wakefulness (Figure 5). Concurrently, moderate-intensity activity increased more substantially in the treatment group (3.2% increase) than in placebo animals (0.7% increase), while high-intensity activity showed minimal differences between groups (0.3% vs. 0.2% increase). This activity profile—characterized by reduced overall activity but improved daytime wakefulness and moderate activity—suggests a shift toward more balanced activity patterns, rather than sedation or lethargy.

**Figure 6:**
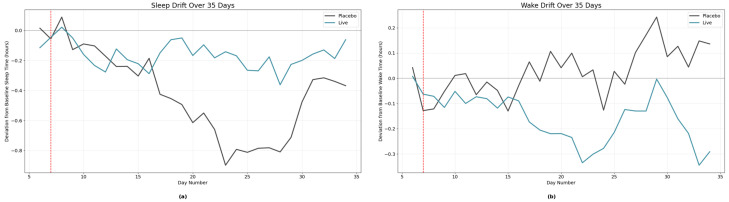
Sleep (**a**) and wake (**b**) time drift over the 35-day study period in placebo (gray) and treatment (green) groups. Treatment dogs exhibited greater stability in sleep onset and wake time after the baseline phase (red line), while placebo dogs showed a progressive shift toward earlier sleep and later waking. These findings suggest that supplementation promoted more consistent sleep schedules and slightly earlier wake times, potentially reflecting improved sleep quality.

In addition to activity levels, the tracker proved instrumental in evaluating alterations in sleep patterns. Minute-by-minute data were used to examine changes in sleep onset during nighttime hours across the trial period, comparing the placebo and treatment groups. Sleep onset was defined as a 15-min interval after 19:00 during which activity levels dropped below a pre-established threshold for light activity, indicative of a stable sleep state. Analysis revealed that dogs receiving the supplement exhibited greater consistency in sleep timing, with reduced variability in sleep onset (Figure 6). Conversely, dogs in the placebo group displayed a significant negative drift in sleep onset, suggesting a progressive shift toward earlier sleep initiation over the course of the study. A comparable methodology was applied to assess waking drift, defined as a 15-min period of activity exceeding the minimum threshold after 05:00. Here, the treatment group demonstrated enhanced stability, with wake times trending slightly earlier, whereas the placebo group exhibited a positive drift, reflecting progressively later wake times. While these findings are limited by the absence of detailed baseline data on individual canine sleep patterns, the combined results suggest that dogs in the treatment group experienced more consistent sleep schedules and slightly earlier waking times, potentially indicative of improved sleep quality throughout the night.

**Figure 7:**
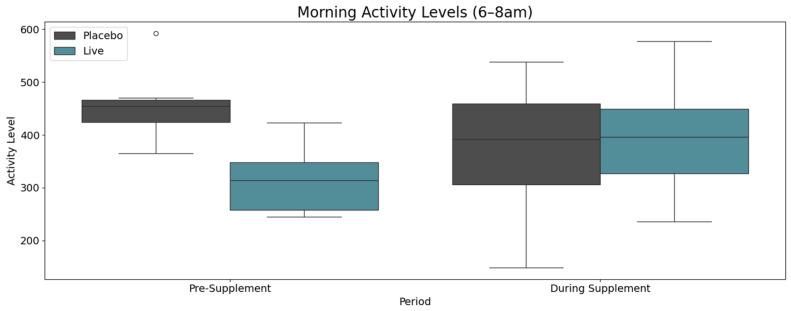
Morning activity levels (6:00–8:00 AM) before and during supplementation. Treatment group dogs exhibited an increase in early morning activity, nearing significance (*p* = 0.075, Wilcoxon signed rank test), suggesting improved wakefulness and readiness for daily activity.

The enhancement in sleep quality is further corroborated by the evaluation of daytime activity levels, which exhibited more pronounced improvements in the treatment group (Figure 7). Between 06:00 and 08:00, spanning the interval from wake times to the departure times of pet caregivers, median activity levels in the treatment group showed a notable increase. This finding reinforces the aforementioned observations and suggests that dogs in the treatment group displayed elevated energy levels during early morning hours, potentially reflecting a heightened state of alertness and readiness for daily activity.

**Figure 8:**
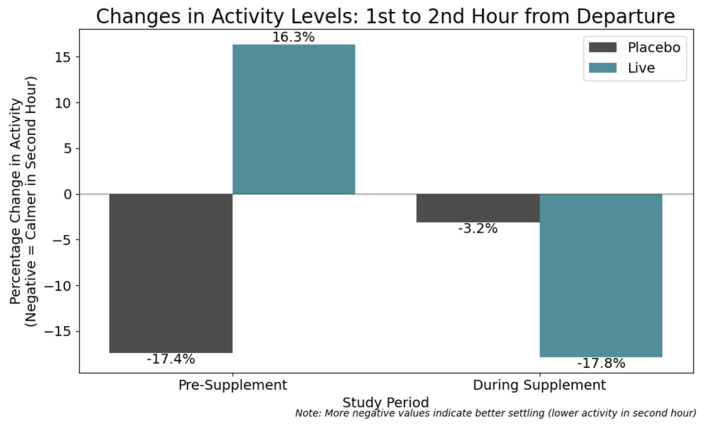
Change in post-departure activity levels (08:00–10:00) between the first and second hours. During supplementation, treatment dogs showed a greater decline in activity (−17.8%) compared to placebo (−3.2%), suggesting improved post-departure calmness and reduced anxiety-related behaviors.

Finally, activity tracker data corroborated findings from C-BARQ results, which indicated reduced anxiety. For dogs with elevated anxiety, it is hypothesized that activity levels increase markedly in the hours following pet caregiver departure due to behaviors such as pacing. To test this, post-departure activity was assessed within a 2-h window (08:00–10:00) following the average caregiver departure time of 08:00. Mean activity levels were calculated for the first (0–59 min) and second (60–120 min) hours, with percentage changes reflecting settling behavior. Dogs in the treatment group exhibited a more substantial reduction in activity during the supplement phase (e.g., −17.8% vs. −3.2% for placebo), suggesting enhanced post-departure calmness. This may reflect a reduction of anxiety-related traits in the treatment cohort following probiotic supplementation (Figure 8).

## 4. Discussion

This is the first randomized, placebo-controlled trial to evaluate the patented GABA-producing strain Lactiplantibacillus plantarum LP815™ for mitigating behavioral issues in home-based companion dogs. The study also pioneers a fully decentralized, multimodal assessment that pairs objective FitBark activity data with owner-reported C-BARQ scores. Although a pilot study previously showed FitBark’s feasibility for behavioral monitoring, it did not test LP815™ or combine sensor and questionnaire endpoints as conducted here [46].

Our findings indicate significant improvements in aggression and anxiety, supported by validated C-BARQ assessments and objective activity tracking. LP815^TM^ treated dogs settled more quickly after owner departure, exhibited decreased daytime activity without lethargy, and demonstrated more consistent sleep patterns with reduced daytime napping. No adverse events were reported across more than 1120 administered doses, supporting the safety profile of LP815^TM^.

As this was the first study evaluating LP815^TM^ in dogs, we adopted an exploratory approach, enrolling dogs with a broad range of behavioral concerns. C-BARQ allowed comprehensive profiling, reflecting the high prevalence and diversity of canine behavioral conditions. Prior studies have shown that behavioral problems are a major concern among veterinarians, with aggression being a leading cause of pet abandonment [47,48,49,50]. Yet, many veterinarians feel ill-equipped to manage these issues, indicating a gap in current clinical practice [51].

Pet-caregiver surveys echo this prevalence. Yamada et al. reported 86% of Japanese households observed at least one behavioral issue [2], while Dinwoodie et al. found 85% of UK dogs had at least one problem [3]. Notably, aggression and anxiety often co-occur, as evidenced by Salonen et al., who found fearful dogs were 3.2 times more likely to show aggression [4]. LP815^TM^ reduced both traits in our study, suggesting the involvement of a shared underlying mechanism.

Pharmaceutical treatments like fluoxetine are common but present challenges. While effective in some cases, SSRIs often have side effects, require weeks to work, and risk serotonin syndrome in canines [52,53,54]. In contrast, LP815^TM^ produced behavioral improvement within 4 weeks, without adverse effects.

Nutritional supplements represent a growing alternative. Diets rich in amino acids and herbal extracts have modulated anxiety markers in dogs, but many lack rigorous controls or long-term efficacy [55,56]. Supplements involving GABAergic pathways, such as omega-3s and magnesium, offer some promise [57]. However, sustained behavioral change likely requires targeting upstream mechanisms—such as enhancing gut microbial GABA production.

LP815^TM^ may function by improving gut microbiome composition, increasing GABA synthesis, and stimulating vagal nerve signaling pathways. Prior studies with GABA supplementation in dogs reported reduced night-time distress and activity [58,59]. However, direct GABA administration may not sustainably elevate host GABA levels, as suggested by human plasma data indicating that GABA has a short half-life and does not accumulate even with repeated dosing [60]. Modulating the microbiota to endogenously produce GABA may provide longer-lasting effects.

Only one other study has explored an *L. plantarum* probiotic (PS128) in dogs with aggression and separation anxiety [61]. Despite a shorter duration (14 vs. 35 days), the outcomes aligned with our results, supporting the behavioral impact of this strain. The authors concluded that PS128 stabilized emotional regulation and decreased the severity of existing behavioral problems compared to the control group. Notably, both aggression and separation anxiety subgroups showed decreased symptom severity, aligning with outcomes observed in our study. This study also employed an abbreviated behavioral evaluation rather than a full assessment. Our use of comprehensive C-BARQ data and a longer treatment duration in our study provides stronger support for the modulation of these behaviors through a microbiome-based intervention.

A novel aspect of our study was improved sleep regularity in treated dogs. While canine-specific literature is lacking, studies in humans and rodents support GABA’s role in regulating sleep–wake cycles [32,61,62]. This points to a potentially new application of GABA-producing probiotics in dogs to support sleep cycle management.

Future studies should refine sleep analysis using tools capable of assessing sleep architecture and stages in dogs. Biomarker tracking (e.g., cortisol, oxytocin, serotonin) and microbiome profiling could further elucidate the mechanisms by which LP815^TM^ works. In our study, we exclusively worked with home-based dogs to better mirror the application of the supplement in a commercial, non-therapeutic setting. Considering the results of this trial, it may be relevant to conduct further research including a veterinarian-in-the-loop to assess whether the use of the supplement reduces veterinarian-administered C-BARQ results as well as other methods of behavioral assessment. Trials focusing on aggressive breeds or dogs with extreme anxiety specifically could validate LP815^TM^’s role in more targeted behavioral therapy. Additionally, considering the proposed mechanism of action of LP815^TM^ via sustained in situ GABA production, it would be interesting to explore the longer term impact of supplementation, both in terms of wash-out period (i.e., how long the supplement has an impact after dosage has ended) as well as whether longer supplementation further improves measured metrics, as found in human cohorts (cite sleep study pre-print). Although the present trial deliberately used an inert maltodextrin placebo to isolate the strain-specific effect of LP815™, the positive outcomes observed argue for a next step that includes an active comparator. Future studies should therefore attempt to evaluate LP815™ head-to-head with established pharmacologic options such as fluoxetine to determine whether the probiotic can match or exceed the clinical benefit of standard therapy while offering a distinct safety and convenience profile. Although type of diet was not an inclusion criterion, it is likely that historical difference in diet may modulate the impact of LP815™ due to it acting within the gastrointestinal tract. Baseline microbiome profile and diet were out of the scope of this research, but may warrant future investigation. Furthermore, although our 4-week intervention reflects prevailing probiotic-trial conventions for microbiome and behavioral outcomes, future studies should extend supplementation to 6–12 weeks to determine whether LP815™’s benefits continue to accrue or plateau over longer-term use, mirroring the timelines observed in the parallel human anxiety study [32]. Finally, further increasing sample size and incorporating genetic validation for breed identification could serve to further strengthen our conclusions in this study.

## 5. Conclusions

In summary, LP815^TM^ shows promise as a safe, effective probiotic intervention for canine anxiety and aggression, with benefits that may reach into sleep and calming. Our results suggest a broader mechanism involving gut–brain axis modulation and underscore the potential of microbiome-based behavioral therapies, as well as the need for continued research into them.

## Data Availability

The datasets generated and analyzed during the study are available from the corresponding author upon reasonable request.

## Supplementary Materials

The following supporting information can be downloaded at: https://www.mdpi.com/article/10.3390/ani15152280/s1, Figure S1: Top 10 Questions with the Biggest Changes (Placebo group, Day 0 to Day 35); Figure S2: Top 10 Questions with the Biggest Changes (Live group, Day 0 to Day 35).
