# Supplementary figures and images for: Efficacy of a Novel Lactiplantibacillus plantarum Strain (LP815TM) in Reducing Canine Aggression and Anxiety: A Randomized Placebo-Controlled Trial with Qualitative and Quantitative Assessment
Source: Animals (Basel). 2025 Aug 4;15(15):2280. doi: 10.3390/ani15152280 (PMC12345577; doi:10.3390/ani15152280)

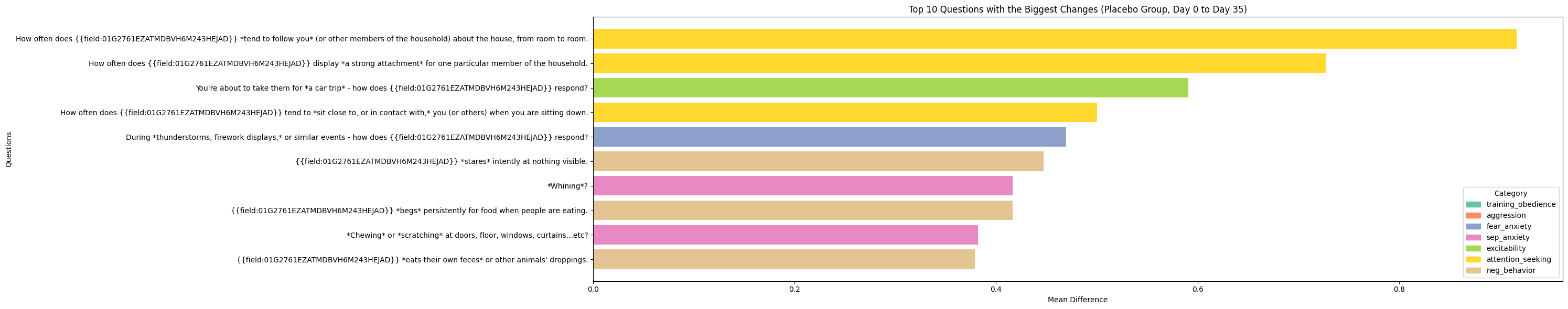

Supplement: Supplementary file 1 [file animals-15-02280-s001.zip › animals-3776972-supplementary figure S1.png]

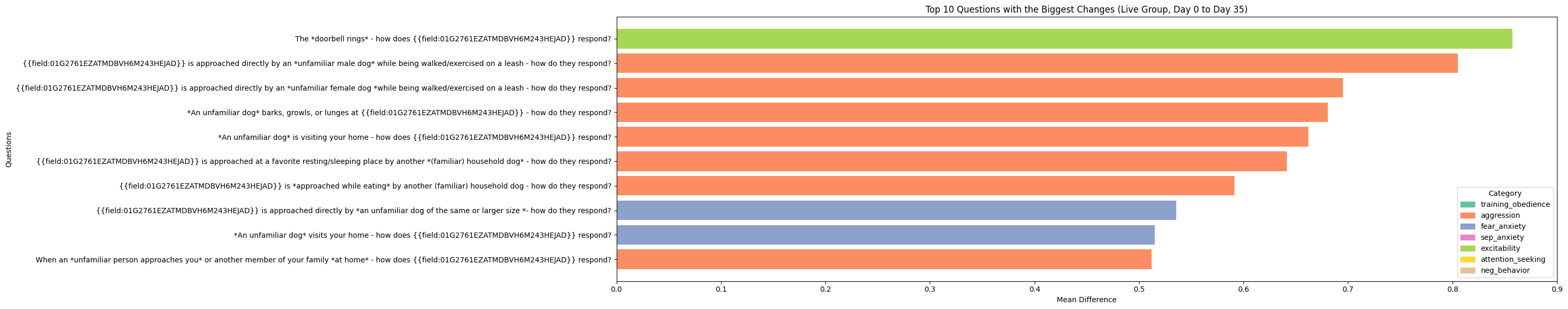

Supplement: Supplementary file 1 [file animals-15-02280-s001.zip › animals-3776972-supplementary--S2.png]
